# Metabolic biomarkers for the differential diagnosis of pancreatic ductal adenocarcinoma vs. chronic pancreatitis

**DOI:** 10.1186/2049-3002-2-S1-P60

**Published:** 2014-05-28

**Authors:** Julia Mayerle, Holger Kalthoff, Regina C Reszka, Beate Kamlage, Erik Peter, Bodo Schniewind, Sandra González-Maldonado, Volker Liebenberg, Christian Pilarsky, Philipp Schatz, Jonas A Schreiber, Ulrich F Weiss, Robert Grützmann

**Affiliations:** 1 Department of Medicine A, Ernst-Moritz-Arndt-University, Greifswald, Germany; 2 Institut for Experimental Cancer Research (IET), Section for Molecular Oncology; UKSH, Kiel, Germany; 3 Metanomics Health GmbH, Berlin, Germany; 4 metanomics GmbH, Berlin, Germany; 5 Clinic and Outpatient Clinic for Visceral-,Thorax-and Vascular Surgery, University Hospital, Carl Gustav Carus, Dresden, Germany; 6 Thermao Fisher Scientific, Biomarkers, Henningsdorf, Germany

## Background

The incidence of chronic pancreatitis (CP) varies between 4 and 23/100.000 in different populations and a tenfold higher prevalence. Current diagnostic tests such as transabdominal ultrasound and CA 19-9 can distinguish between pancreatic cancer (PDAC) and chronic pancreatitis (CP) in only about two thirds of patients. CA19-9 has been reported to discriminate between pancreatic cancer patients and healthy controls with a sensitivity of 0.80 (95 % CI 0.787-0.83) and a specificity of 0.80 (95 % CI 0.78-0.82). Therefore more sensitive biomarkers for the early detection of pancreatic cancer would be urgently needed. Our aim was to identify a panel of plasma metabolite biomarkers for this diagnostic purpose.

## Materials and methods

For a case-control study, 914 subjects were retrospectively recruited from three tertiary referral centers with either pancreatic cancer (n=271), chronic pancreatitis (n=282), liver cirrhosis (n=100), or healthy as well as non-pancreatic disease controls (n=261). An initial exploratory study (n=201) was followed by a two-center identification study (n=474; training set) and a third validation test set (n=239). Metabolomic profiles of plasma and serum samples were generated using the comprehensive metabolome platform including lipidomics ((MxP® Broad Profiling, MxP® Steroids, MxP® - gas chromatography-mass spectrometry (GC-MS) and liquid chromatography-tandem mass spectrometry (LC-MS/MS) ) identifying 477 metabolites.

## Results

A consistent multimarker 10-metabolite panel (including sphingomyelin and ceramide) was identified by the Elastic Net algorithm providing an area under the curve (AUC) of up to 0.96 [95% CI 0.93-0.98] (pancreatic cancer vs. chronic pancreatitis). With a fixed sensitivity of 88% and an estimated incidence of 1.95 for pancreatic cancer a test specificity of 93.8% [95% CI84-98%] for the training set and of 91.3% [95% CI 76-94%] for the validation (test) set was reached with a negative predictive value (NPV) of 99.75% [95% CI 99.7-99.8%]. Pancreatic cancer can not only be detected but also distinguished from chronic pancreatitis when still in a resectable (i.e. early) stage.

## Conclusion

These results indicate that a plasma metabolite biomarker panel including important metabolites identified by the lipidomics platform can be used to accurately distinguish between pancreatic cancer and chronic pancreatitis. Sphingolipids are characterized by the presence of a particular aliphatic amino alcohol, sphingosine. Cleavage of sphingomyelins by sphingomyelinase generates ceramide, which promotes apoptosis, cell cycle arrest and cellular senescence. It also permits the development of a promising diagnostic plasma biomarker assay for the detection pancreatic cancer in high risk cohorts.

